# Implications in Halotherapy of Aerosols from the Salt Mine Targu Ocna—Structural-Functional Characteristics

**DOI:** 10.3390/healthcare11142104

**Published:** 2023-07-24

**Authors:** Mihaela Orlanda Antonovici (Munteanu), Ioan Gabriel Sandu, Viorica Vasilache, Andrei Victor Sandu, Stefanita Arcana, Raluca Ioana Arcana, Ion Sandu

**Affiliations:** 1 Doctoral School of Geosciences, Faculty of Geography and Geology, Alexandru Ioan Cuza University of Iași, 22 Carol I, Blvd., 700506 Iasi, Romania; mihaelamunteanu18@yahoo.com; 2 Faculty of Material Science and Engineering, Gheorghe Asachi Technical University, 64 Dumitru Mangeron Blvd., 700050 Iasi, Romania; sav@tuiasi.ro; 3 Romanian Inventors Forum, 3 Sf. Petru Movila St., L11, 3-3, 700089 Iasi, Romania; 4 Arheoinvest Center, Department of Natural and Exact Sciences, Institute of Interdisciplinary Research, Alexandru Ioan Cuza University of Iasi, 22 Carol I Blvd., 700505 Iasi, Romania; viorica_18v@yahoo.com; 5 Academy of Romanian Scientists, 54 Splaiul Independentei St., Sect. 5, 050094 Bucharest, Romania; 6 Doctoral School of the Faculty of Medicine, Grigore T. Popa University of Medicine and Pharmacy, 16 Universitatii Str., 700115 Iasi, Romania; arcana.stefan@gmail.com (S.A.); arcana.raluca@umfiasi.ro (R.I.A.); 7 Clinical Hospital of Pulmonary Diseases, Str. Dr. I Cihac, Nr. 30, 700115 Iasi, Romania; 8 National Institute for Research and Development in Environmental Protection, 294 Splaiul Independentei, Sector 6, 060031 Bucharest, Romania

**Keywords:** Aitken particle, Solion, salt micro-aeroanion, glomerular cluster, coordinated aquatemplation, low packing salt micropolyhedra, water pentahydrols, halochambers, prevention, therapy

## Abstract

The paper presents the evolution of the concentration level for four particle size groups of microaerosols (1.0, 2.5, 4.0 and 10.0 µm) in correlation with the microclimatic characteristics (temperature, humidity, lighting, pressure and concentration in CO_2_ and O_2_) in three active areas of the Targu Ocna Saltworks, currently used in treatments with solions (hydrated aerosols): in the vicinity of the walls of the old mining salt room, where there is a semi-wet static regime (SSR); in the transition area between the old rooms of exploitation with the semi-wet dynamic regime (DSR); and in the area of the waterfall and the marshy lake with the dynamic wet regime (DWR). The first and last halochamber are the ones recommended for cardio–respiratory, immuno–thyroid and osteo–muscular conditions, as well as in psycho–motor disorders. Based on questionnaires carried out over the course of a year, between 1 September 2021–31 August 2022, in two periods of stationing/treatment: a cold one (15 September 2021–15 December 2021) and a warm one (1 May 2022–30 July 2022), correlated with the data from the Salina medical office, achieved the profile of the improvement rate of the patients’ ailments depending on the type of treatment (working regime in halochambers). These studies have allowed the optimization of the treatment conditions in the artificial surface halochambers in order to reduce the stationary period and optimize the treatment cycles.

## 1. Introduction

Currently, the effect of the environment in the salt mines is known, where along with the saline aerosols, the internal energy field of the rock salt block has a beneficial role on the human body. The therapeutic effect of the halosalt mining climate and the Slatina Springs (natural salt solution/brine) is given by the set of physico–chemical and microclimatic factors/agents, which manifest as an integrated system, with complex effects on the human body [1,2,3,4,5,6,7,8,9,10,11].

The therapeutic role of the saline environment has been recognized since the 19th century, following some observations, noting the absence of chronic bronchitis and asthma in the miners of the Wieliczka salt mines, as well as the rapid healing of these diseases in the new employees, when the improvement of asthma attacks in chronic patients was observed [1,2,3,4,5].

In Romania there are many deposits of rock salt, which have been exploited since ancient times (Figa, Bistrita Nasaud county), over time, they became sources of salt for domestic use (food, traditional household uses, such as: preserving vegetables and of meat, leather tanning, etc.), but also with many industrial applications (from chlorosodium industries, to heat treatments in metallurgy). Many mining areas, with underground or surface mining, are still active today. Most of them, in the last 50 years, have developed balneo-climatic treatment systems. Of these, in the Moldova area, in the Eastern Carpathians, there were two rock salt mines, the one at Cacica, from Suceava county, and the famous Targu Ocna (Tg. Ocna) Salt Mine, from Bacău county [9,10,11,12,13,14]. The last one is located in the Trotuş Valley, in a special natural setting, not far from the well-known balneo-climatic resort Slănic Moldova. In recent years, Salina Tg. Ocna developed in parallel with the activity of exploitation, processing and marketing of salt, a field that attracts the public from all corners of the world, through the activity of tourism and halotherapy, which includes, in addition to a series of old salt exploitation rooms, very large aerosol generating surfaces and a number of objectives sought for treatment, training and/or leisure, of which the most requested is the salt water lake and the waterfall, where due to the very high hygroscopic humidity there are high concentration levels in solions (microaerosols hydrate), which provide ideal atmospheres for salinotherapy. Among other attractions of Salina, we mention the “Saint Varvara” church, located 240 m underground, the only one in Europe built entirely of salt, and the Salt Museum, which offers information about the genesis, the evolution of the exploitation and processing of salt and other artifacts regarding its therapeutic virtues, which includes interesting exhibits related to underground mining and therapeutic activity [12].

Natural halochambers (rock salt mines) and artificial ones, on the surface, contain anhydrous, semi-hydrated and hydrated aerosols. The last two types are layered microclusters of aquated aeroanions. They are reformed by coordinative aquatempering of NaCl nonpolyhedra, with low packing, under the influence of water pentahydrol from humid atmospheres (>80%). Through superstructure, layer by layer, clusters are formed with the formula [x(NaCl)_2n_⋅y(H_2_O)_5_]_(aq)_^q−^, where n > 2, and x and y take values corresponding to some glomeruli with diameters between 0.5 and 50 µm, which have a subunit electric charge q− on the surface, which gives the solion the behavior of a negative spherical aeroion [10,11,15,16,17,18,19,20,21,22].

Depending on the type of source, the activity of rock salt microparticles, respectively their life time and the environmental conditions, solions and other saline aerosols in the atmosphere of natural or artificial halochambers present a three-modal Gaussian dimensional distribution and a somewhat regular concentration (constant in time and space), as a result of the difference between the speed of production and that of extinction or loss, attributed to processes of condensation, coagulation, peptization, electroneutralization, sedimentation (destabilization), etc. [18,19,20,21,22,23,24].

According to our studies [15,16,17,18,19,20,21,22], at a hygroscopic humidity higher than 80% of the total anhydrous microaerosols generated by mobile sources based on cartridges with diaphragms containing NaCl granules obtained by recrystallization, which can reach the maximum level in the halochamber of 12 mg/m^3^, more than 90% of the Aitken particles (<10 µm) are converted by structural reformation into solions, on the other hand, anhydrous saline aerosols with granulometry between 50 and 500 µm have a conversion degree below 20%.

For therapeutic environments, natural or artificial halochamber type, submicron, gaseous microdispersions are used in the form of solions (hygro- or aquo-aerosols) or partially moistened (semi-hydrated) aerosols. For ambient environments, which impose the “clean air effect”, semi-hydrated aerosols with a negative surface charge are primarily used. They destabilize, through electroneutralization, positively charged aerosols, such as those resulting from combustion/pyrolysis (for example cigarette smoke), as well as microdispersions resulting from human breathing, the metabolism of certain fungi or molds, etc. [18,19,20,21,22,25,26,27,28,29,30,31,32].

Semi-hydrated saline solutions and aerosols from natural or artificial sources, depending on their practical applications, must have an activity, characterized by a certain level of concentration, a determined period of life and a range of granulometry rigorously controlled in time [15,16,17,18,19,20,32,33,34,35,36,37,38].

Table 1 presents the seven medical fields of application studied in recent years within our collective and by other research teams around the world.

The healing properties of halotherapy are generated by a synergism between hydrated saline aerosols/solions and microclimate factors. Hydrated saline aerosols dispersed in the saline atmosphere are inhaled through the respiratory tract or absorbed transcutaneously. Depending on the diameter of the particles in the Aitken group, by inhalation they act at different levels of the respiratory tract by accelerating the mucociliary clearance and optimizing the pulmonary surfactant. It is known that halotherapy influences the symptoms of the respiratory system by improving the act of breathing and the patient’s feeling of liberation as a result of increasing cough productivity and lung clearance, reducing infectious pus of the respiratory system due to the bacteriostatic effect and, last but not least, reducing sinus edema [59]. The same effects are in the cases of cardio and immuno–thyroid conditions. Effects of improving human performance have also been highlighted. Thus, physical activities carried out in order to maintain or restore health have a beneficial effect on the body if they are carried out in an environment with saline aerosols, because this exposure over time leads to an increase in the ventilation power of the respiratory system, the amplitude of respiratory movements increases simultaneously with the decrease in their frequency, when the functional capacity of the circulatory system increases (due to the strengthening of the heart) [18,60,61,62].

The researches of recent years regarding the theoretical and applied aspects of saline aerosols have substantiated two new directions of study: the characterization of the hydrated anionic microstructures of the stable solions in the atmosphere of artificial (surface) halochambers and, respectively, the minimization of the stationary time and the optimization of the treatment cycles for the multiple their applications, starting from creating environments with clean air, up to those regarding the prevention and treatment of cardio–respiratory, thyroid and musculoskeletal disorders, culminating in the improvement of human performance in children, the elderly and those who work in harsh conditions or with high effort. Currently, it is known that halotherapy amazingly relieves: severe asthma, respiratory diseases, rhinitis, pharyngitis, otitis, tonsillitis, sinusitis, colds, coughs, allergies (this therapy is also indicated for children), various skin problems, eczema, dermatitis, psoriasis, viral infections, insomnia, and anxiety, restoring the immune system [63].

In the last three years, the COVID 19 pandemic has affected a considerable percentage of human subjects, which required a reorientation of home, outpatient and inpatient treatment protocols for a longer period.

In the past, halotherapy involved a visit to a salt pan, but nowadays there are artificial surface halochambers both in polyclinics and hospitals, as well as in hotels with swimming pools and SPAs [1,2,3,4,5,12,13,14,15,18,25,26,27,55,56,60,61,62].

The paper considers the determination of the concentration level for four granulometric groups of microaerosols (1.0; 2.5; 4.0 and 10.0 µm) and their life time, in correlation with the microclimatic characteristics (temperature, humidity, lighting, pressure and concentration in CO_2_ and O_2_) in three active areas of the Targu Ocna Saltworks, currently used in underground treatments: in the vicinity of the walls of the old exploitation room, gem salt (semi-wet static regime), in the transition area between the old exploitation chambers (regime dynamic semi-wet) and in the area of the waterfall and lake with slush (dynamic wet regime).

Based on the data from the records of the medical office at the Salina level and the questionnaires carried out on human subjects, taking into account one of the three specific activities for therapy, training and rest/visit, the profiles were developed with the evolution of the rate of improvement of the conditions and improving human performance. The questionnaires were carried out on patient segments, in two different periods of hospitalization as climatic environment (winter/summer) and for specific cycles of treatment and training. Each time, the cases of contamination with the COVID virus and the moment when the test became negative were taken into account. These studies allow the improvement of the treatment conditions in artificial surface halochambers and implicitly the reduction of the stationary period to the minimum and the optimization of the treatment/training cycles.

## 2. The Experimental Part

### 2.1. Monitoring the Three Halochambers

**Figure 1:**
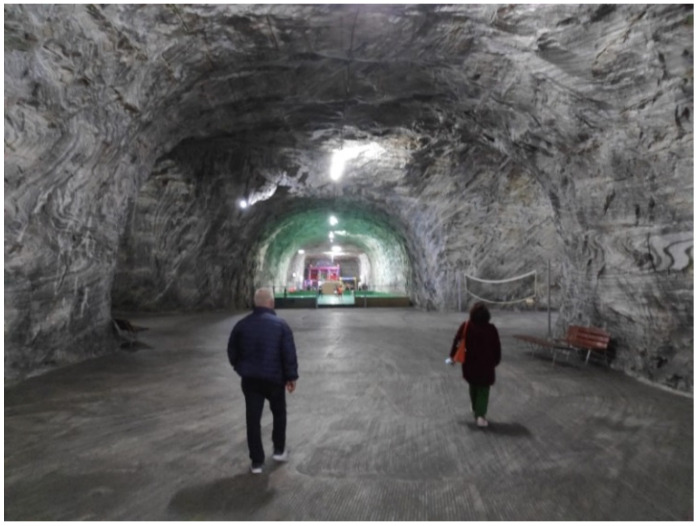
Old exploitation room. Halochamber with semi-wet static mode. Area with training and fitness equipment (acquisition of microclimate data at the level of the salt wall).

**Figure 2:**
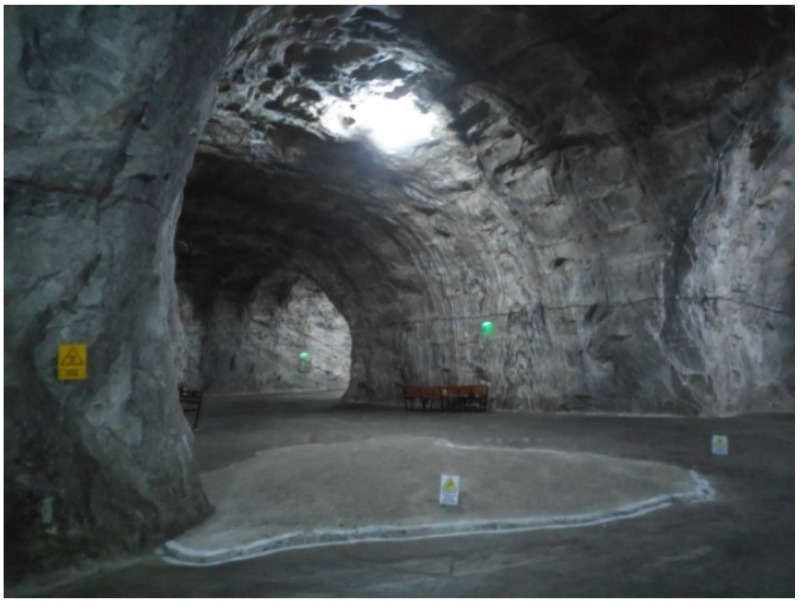
Corridor between the old exploitation rooms. Halochamber with semi-wet static regime (retrieving microclimate data from the center of the transition corridor).

**Figure 3:**
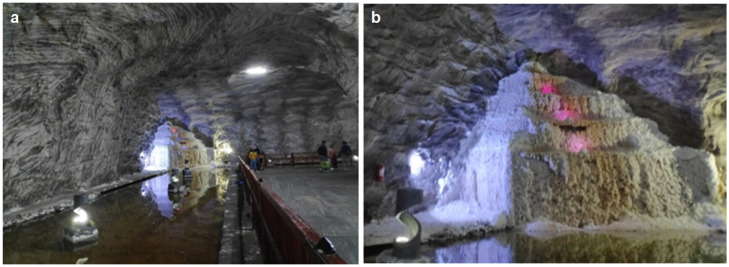
Saltwater lake and waterfall: (**a**) halochamber with wet dynamic mode; (**b**) detail with the waterfall area.

In order to determine the microclimate factors and the physical–chemical characteristics of the treatment environment in the Targu Ocna Saltworks, three areas with different specificity for the microclimatic conditions were chosen: an old exploitation room, with data collection at the level of the salt wall—semi-wet static regime (Figure 1); the passageway between the old exploitation rooms, which present very weak air eddy currents, with the retrieval of the data from the center of the transition zones—semi-humid dynamic regime (Figure 2) and the room with the salt water lake and the waterfall, with the retrieval of the dynamic microclimate data with high humidity—wet dynamic regime (Figure 3).

The three areas of acquisition of the physico–chemical microclimate characteristics of the treatment environment were indexed, as follows: static semi-humid regime (SSR), dynamic semi-humid regime (DSR) and dynamic wet regime (DWR). The walls of the old exploitation rooms contain at least 97.5% NaCl.

The determinations were carried out over the course of a year, between 1 September 2021 and 31 August 2022, in two periods of stationing/treatment: a cold one (15 September 2021–15 December 2021) and a warm one (1 May 2022–30 July 2022).

### 2.2. Methods and Techniques for Determining Microclimate Characteristics and Aerosols from the Three Halochambers

The study of the internal and external environmental factors, respectively, of the physico–chemical characteristics of the microclimate of the treatment environment in Targu Ocna Salina and from the outside was carried out by means of instrumental methods that allowed the determination of the air temperature (Xiaomi MIIIW digital thermometer, Beijing, China), the relative humidity of air (digital hygrometer Xiaomi MIIIW, Beijing, China), lighting (digital luxmeter UT383 Uni-T, Uni-Trend Technology Co., Ltd., Dongguan, China), pressure (barometer TFA, TFA Dostmann GmbH & Co. KG, Wertheim-Reicholzheim, Germany), concentration in CO_2_ (TESTO 315-3, West Chester, PA, USA) and O_2_ (Greisinger GMH 3692, Remscheid, Germany). Additionally, to determine the concentration level of the aerosols, a DUSTTRACK Aerosol Monitor 8532 (Shoreview, MN, USA) type portable particle counter was used, which allowed the measurement of aerosols for the size fractions PM1.0, PM2.5, PM4.0, and PM10.0, and which had the ability to record data in a single point through manual or programmable functions. The aerosol concentration range recorded by this device varies between 0.001 and 150 mg/m^3^. The technique is one of great precision, the recorded values can be stored and then detailed through the graphic presentation of the aerosol concentration variation over time. For each area, four determinations were made to measure the concentration level of aerosols in the four standardized granulometric groups: 1.0, 2.5, 4.0 and 10.0 µm.

The first halochamber is an old rock salt extraction room, with a single access road, the second a transitional passage between old extraction rooms and the third, the most frequented, with a waterfall and brine lake (Slatina). An analysis area was positioned in each of the three halochambers, allowing the reproducibility and lack of fluctuations of the analytical data, the coherence between the coexistence and the corroboration of the measurement techniques. In the first halochamber, the data were measured at the level of the wall opposite the entrance (SSR); in the second, at the center of the access lane between the halochambers (DSR); and in the third, at the wall next to the waterfall at the level of the surface of the Slatina Lake (DWR). Before determining the level of solions for the four granulometric groups (1.0, 2.5, 4.0 and 10.0 µm), air temperature, relative humidity, lighting, atmospheric pressure, CO_2_ and O_2_ concentrations were measured. We also took into account a series of characteristics of the saline previously determined by the administrative and medical staff, with reference to the air currents in each halochamber, the pH of the air, radioactivity, ozone concentration, etc., which were used in the substantiation of some experimental aspects related to the physico–chemical characterization of the climatic environment and the solions.

### 2.3. Evaluation of the Profile of Human Subjects Based on the Results Obtained from the Therapy/Training Activities in the Three Halochambers

**Figure 4:**
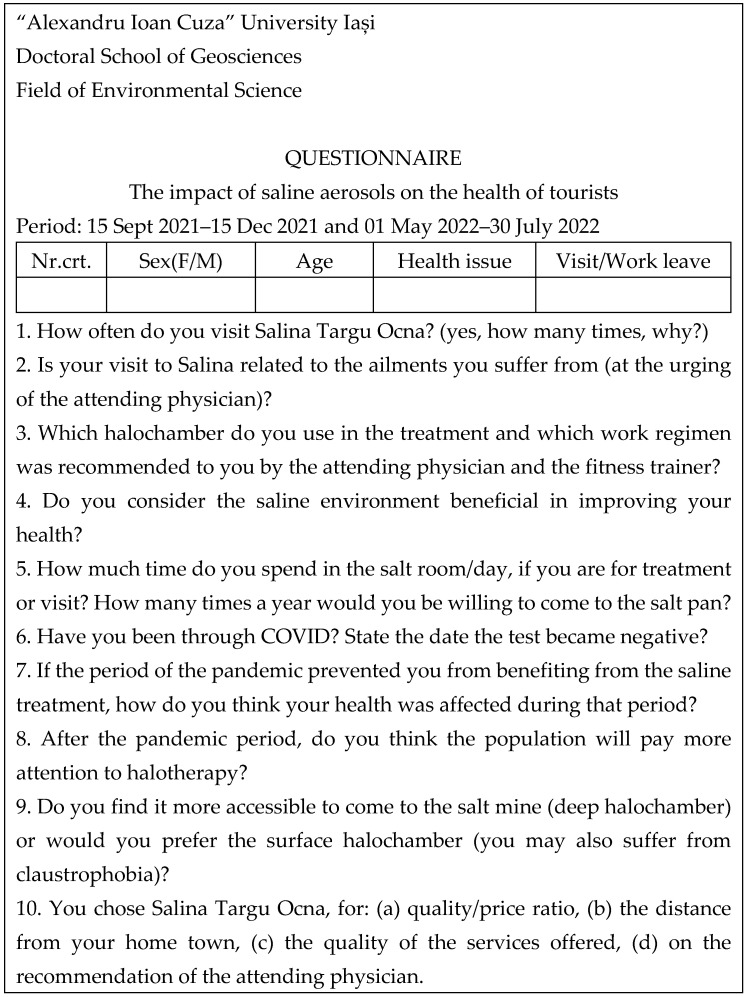
The model of the questionnaire registered for the two analyzed periods (15 September 2021–15 December 2021 and 1 May 2022–30 July 2022) the patients, athletes and tourists present in Salina Tg. Ocna.

Using the data on the patients from the period under study, who performed cures/therapy sessions/treatment in Salina, monitoring based on a questionnaire with 10 items/questions (Figure 4) made on patient segment, in two periods of stationing/treatment (15 September 2021–15 December 2021 and 1 May 2022–30 July 2022), the profile of the patients regarding the improvement rate of the main types of conditions frequently encountered here was made and the impact on the patients of the solions (hydrated saline aerosols) from the dynamic humid halochamber was evaluated (DWR). The therapy regimens were co-assisted with the training regimens at the level of each halochamber.

The questionnaire was approved by the board of PhD supervision and validated by a team of PhD and medical doctors specialists in the field. 

The treatment program in the three halochambers includes a wide range of common exercises, such as: rest, general medical and respiratory gymnastics, fun and sports games (chess, tennis, basketball, etc.), walks, moderate training runs at effort, health education programs and others, which were recommended by the attending physician and the fitness trainer [64,65,66].

Upon their recommendation depending on the condition, but also on the wishes of the patient, athlete or tourist, a daily activity (work) regimen was established for each person, consisting of [64,65,66]:

- *medical, artistic-sports, rhythmic and respiratory gymnastics;*
- *fitness, jogging and isometric exercises;*
- *strength training exercises at least twice a week;*
- *aerobic training with high-intensity exercises (HIIT), alternating short periods of vigorous exercise with short ones, but at a slower pace;*
- *autogenous relaxation, carried out individually, after its correct appropriation by the patient/athlete;*
- *active rest by walking slowly or walking along the paths that cover the three halo chambers, respectively by reading or other sports (chess, rummy, billiards, etc.);*
- *yoga, pilates and meditation are recommended especially in the morning.*

Of these exercises, those for *circuit training* involve repeating without breaks or with very short breaks, for those that work the whole body in a single session, such as fitness or other intense or less intense physical exercises. With this kind of circuit training, both the specific benefits of cardio exercises and those of strength training are obtained, developing muscles and physical strength [64,65,66].

Often in effective training, which can last under 30 min, even without using any type of equipment, it was the ideal choice for those who wanted a quick workout or who did not require a lot of space at their disposal. For example, a first exercise might work the chest using a pair of *dumbbells,* then move on to the *leg press*. In this way, the upper body muscles rest while the lower body is worked.

Additionally, an optimal training used included combinations of exercises related to body weight or different fitness machines, respectively, for various sports or medical training. The type of exercise chosen was found to be less important; the key to circuit training is to select the exercises in such a way as to work the entire body in one session.

Training methodologies used for endurance activities such as running and complex exercise have taken into account: specific body building and fitness characteristics, biological and behavioral variables, mental and nutritional status [64,65,66].

In carrying out the work regime for a patient or groups of patients, according to sports practices, the intensity of the exercises was expressed by: movement speed, in the case of cyclical sports; the number of actions in the unit of time in the case of performance sports; the number of technical–tactical procedures per time unit in sports games; the number of exercises per time unit in medical gymnastics, artistic sports and rhythmic gymnastics;

In establishing a well-dosed effort, the following factors were taken into account [64,65,66]:

*a.*  *the intensity of the exercise—the degree of demand compared to the maximum capacity:*
*b.*  *the duration of the effort,*
*c.*  *the length of breaks between repetitions*
*d.*  *the nature of rest*
*e.*  *volume of effort or number of repetitions.*

In this sense, the survey involving the questionnaire in Figure 4 was developed in such a way as to allow determining the impact of hydrated or semi-hydrated saline aerosols from the three halochambers on various types of ailments, but also on physical and intellectual performance in humans.

In the case of training, a sheet was used highlighting the work regime for each patient, including the conditions and the way to approach the training. Data on the evaluation of treatment profiles in correlation with periods of work, continuous or cyclic, for the cases of therapy, rest/recovery and training were used as a basis for assessment. The questionnaire highlighted a series of aspects related to the evolution of the treatment of patients or, in the case of athletes, the results obtained through training to improve physical or intellectual performance. Additionally, the observance of the working regime and the halochamber used were taken into account.

Special attention was given to people who have been sick with SARS-CoV-2 and needed recovery. In this sense, the evolution of therapy or training exercises in the saline was followed in most cases, which improved their health and physical and intellectual performance.

Thus, in the periods of 15 September 2021–15 December 2021 and 1 May 2022–30 July 2022, on the groups of people/subjects who carried out therapy or training activities, alongside those who came to visit the Salina Tg. Ocna, there were carried out surveys, being monitored daily at the entrance and exit from Salina. Starting from the idea that during this period, with the maximum, for the SARS-CoV-2 and Omicron pandemic, the population was isolated by quarantine, special attention was paid, for the people who were contaminated, to the period after the negative test, which allows highlighting the segment of individuals who have been isolated from the rest of the visitors and those who suffer from other ailments, respectively, to determine the stage of the disease and to reduce the risk of infecting other people.

The questionnaires allowed the identification of some segments of people, grouped by male and female gender, each group being analyzed in two age classes: under 35 years and over 35 years.

The people who required aerosol therapies were grouped according to the following conditions: cardio–respiratory, osteo–muscular, psycho-neuro-motor and immuno–thyroid, and separately analyzed the groups/groups that performed training to increase physical performance or intellectuals and those who are visiting.

The exercise program and working conditions were determined by the attending physician or other specialized personnel, such as fitness trainers. As work duration, the regime of 2, 4, 6 or 8 h was taken into account, with breaks of stationary or moderate effort of 10 to 30 min, and as exercises, depending on the purpose of halotherapy: medical or respiratory gymnastics, exercises with gradual effort, running at different speeds and other physical exercises recommended by the therapy.

## 3. Results and Discussion

Table 2 shows the values of the microclimate and physico–chemical characteristics of the treatment environment in the three halochambers of the Targu Ocna Saltworks.

Based on the results obtained, it was estimated that the bioclimate of Salina Tg. Ocna was characterized by a mild thermal discomfort, due to the cooling effect (corrected by a movement program and appropriate clothing), with a moderate hypotonic skin stress index and a balanced pulmonary stress index, thus, a sedative prevention and treatment bioclimate.

In the rock salt mines, benefiting from very large contact surfaces between the moist air and the walls, the atmosphere of the halochambers was fed permanently and uniformly, naturally, with semi-moist saline aerosols, through the controlled action of the pentahydrols on the dynamic micropolyhedra, with low packing, results by superficial saline fluorescence processes. It should not be forgotten that, over time, these surfaces were continuously covered with biofilms resulting under the influence of the anthropic factor, but also of some salt-resistant microorganisms—aspects that reduce the generation activity, to the detriment of those on the left [18,60,61,62].

Table 3 shows the concentration level of the solions for the four granulometric groups: 1.0; 2.5; 4.0 and 10.0 µm, determined in three areas selected following some preliminary determinations as having specificity differentiated by concentration levels.

The data in Table 3 were taken from plots of aerosol concentration versus time recorded with a DUSTTRACK Aerosol Monitor 8532 portable particle counter for a period of one second.

It was noticed to begin with, that the measurements made in the area of the wall opposite the entrance of the halochamber (SSR) from an old exploitation of rock salt (1952–1970), where the speed of the air current was very low, below 0.01 m/s, the temperature of the environment something higher, around 13.1 °C and 72% lower relative humidity (sufficient for the formation of solions from Aitken-type aerosols), the concentration level in solions for the four granulometric groups were very close in value, being between 0.030 and 0.047 mg/m^3^. In contrast, in the transition halochamber (DSR), where compared to the first zone (SSR), the average temperature was lower (12.8 °C), the average relative humidity a little higher (74%), and the average current speed of slightly higher air (0.03 m/s). Here, an increase in the solion concentration level was felt, between 0.032 and 0.059 mg/m^3^. The last area selected for determinations was the waterfall halochamber and slough lake (DWR), which had the lowest average ambient temperature (12.3 °C), high relative humidity (93%) and slightly higher (0.04 m/s), compared to the DSR area. This was due to the presence of eddy currents of air rising from the surface of the lake to the ceiling of the halochamber. In this halochamber, the highest level was achieved in solions, between 0.046 and 0.117 mg/m^3^. The first halochamber and the last were the most indicated by therapists, for periods of stay between 2 and 8 h, with treatment cycles of 5 to 10 days, depending on the condition or purpose.

The data related to the stationary period and the treatment regimen, known and displayed in each halochamber with free access, correlate very well the levels of solions, with the physico–chemical characteristics of the microclimate in the halochambers and those of the solions.

The halo cameras from Salina Tg. Ocna offered a comfortable climate, with a fairly high concentration of carbon dioxide (0.06–0.08%) and normal atmospheric oxygen (20.8–21.1%), which stimulated and revitalized the respiratory center, and the low concentration of ozone decreases buniolar smooth muscle spasm. It should be noted that the low pH of the air in the halochambers (4.2–4.5) provided a bacteriostatic effect.

Moreover, the absence of air pollutants and allergens (molds and pathogenic germs), the slightly acidic pH of the air and the presence of Na, Ca, Mg etc. ions, along with the degree of air purity, which was high both in summer and in winter, offered Salina Tg. Ocna, as already said, a weak thermal discomfort.

The need to travel to Salina had led in the last 40 years to the creation of reliable, artificial (surface) halochambers, with flexible structural-functional characteristics and adaptable to the purpose pursued in the treatment. They enabled optimal treatment conditions to be achieved by optimizing the operating regime (static or dynamic), with predetermined levels of concentration in the solution, microclimate parameters and other physico–chemical and microbiological characteristics.

Through the detailed study of the structural-functional characteristics of a natural halochamber, artificial surface models could be constructively developed, with destinations from a single application to those with multiple uses. Today, it is known that artificial halochambers must keep the atmospheric pressure as close as possible to the external one (±5 mmHg), the relative hygroscopic humidity between 75 and 96%, and the illumination between 80 and 120 lux, areas in which the activity of solions, in static or dynamic generation mode, is kept constant. In order to achieve the most efficient transfer to the mucous membranes and integuments and to achieve increased relative densities of hydrated, solion-type aerosols, but also to ensure thermal comfort conditions, an optimal temperature range between 12 and 16 °C was recommended.

### Evaluation of the Profile of Human Subjects who Underwent Therapies and Training in the Halochambers of the Salina

Table 4 and Table 5 show, on the three groups of activities (therapy, training and visit) carried out in Salina in the periods of 15 September 2021–15 December 2021 and 1 May 2022–30 July 2022, the profile of human subjects (weight, %), by sex and age groups. In Table 4, the data were correlated with the duration of the activities in the three halochambers; on the other hand, in Table 5 the data are presented on five groups of conditions, one training group and one of visitors (tourists), who performed specific activities in the three halo cameras recommended for therapy sessions, training and visits to Salina.

The data in Table 4 highlighted the different distribution of the profile of human subjects who participated in the periods of 15 September 2021–15 December 2021 and 1 May 2022–30 July 2022 in activities in Salina Tg. Ocna, as follows: for treatments, 72.99%; for training, 7.59%; and, respectively, for rest or visit, 19.42%, highlighting those contaminated with COVID and who had a negative test confirmed at least 30 days ago. Additionally, the duration of the cycles, in minutes, carried out in the three halo cameras was highlighted, which vary as follows: SSR between 1 and 5 min, DSR 3–30 min and DWR between 2 and 20 min, considering that during the training sessions used cyclic regimes with short breaks of 5–10 min in the SSR and DSR halo cameras, and in the DWR halo camera the breaks are a simple pass lasting a maximum of 3 min.

In Table 5, data were presented as the 3 applications regimes (SSR, DSR, DWR) from the Tg. Ocna salt mines (treatments, trainings and visit/leisure) for various types of affections in order to have a general view on the influence of solions (hydrated aerosols).

Table 5 presented the profile of human subjects by the type of activity (treatments, physical training, visits/tourism), with the total highlighting of those previously contaminated with COVID, having a negative test obtained in the last 30 days (indexed with the letter a), those with chronic conditions (indexed with b), those with chronic conditions contaminated with COVID, having a negative test obtained in the last 30 days (indexed with the letter c). The working regimes were indexed for periods of 3–5 min with the letters d and for periods of 5–10 min with e. The therapy cycles for the five conditions under consideration were performed in the DWS halochamber, which was recommended for this purpose. The other two halo cameras were only used for a simple visit in the form of a visit during the breaks between treatment cycles. The working regime in the DWS halo camera was recommended based on previous research [1,2,3,4,5,6,7,8,9,10,11,13,14,15,16,17,18,19,20,21,22].

The last (DWR) was recommended for therapy; from here were also the values presented, the rest being only for transit or visit. Patients with chronic conditions showed an improvement rate (with red color).

The Table 6 presents the disease improvement rate in percentage. From the total cases (100%) only 72.99% were therapy cases and these were distributed by age, sex and conditions.

The data in the table were calculated percentages out of the number of eligible cases.

Table 6 highlighted, for each condition, the share in percentage (%) and the evolution of the improvement of the conditions (indexed in red color), with their reproduction by the rate of improvement by sex and age groups (indexed in blue color), when it was highlighted that female subjects were more responsive to the aerosol treatment than males. Thus, in the COVID recovery therapies, the improvement of cardio–respiratory and immuno–thyroid conditions, the improvement rate had about the same differences between men and women; on the other hand, in psycho–neuromotor recovery in men, the effect of solion therapy is stronger than in women.

## 4. Conclusions

Based on the results regarding the generation of semi-hydrated saline aerosols and those in the form of solions (glomerular microclusters, with concentric stratification), obtained by coordinative aquatemplation with pentahydrols of water of NaCl micropolyhedra, with low packing of several tens of ion pairs, s- studied three of the halochambers from the Targu Ocna salt pan used in various therapies, especially for COVID, cardio–respiratory, immuno–thyroid, neuro–psychomotor and osteo–muscular conditions.

From the analysis of the profile of human subjects, by gender and age group, correlated with the duration of activities in the three halochambers, evaluated by five groups of conditions, one group of training and one of visitors (tourists), who performed specific activities for therapy sessions, training and visiting in Salina, it emerged that 72.99% were subjects for treatments, 7.59% for training and, respectively, 19.42% for rest or visit, of which 15.64% were contaminated with COVID and had a negative test confirmed at least 30 days prior. Additionally, the duration of the cycles, in minutes, carried out in the three halo cameras was highlighted, which varied as follows: SSR between 1 and 5 min, DSR 3–30 min and DWR between 2 and 20 min, considering that during the training sessions used cyclic regimes with short breaks of 5–10 min in the SSR and DSR halo cameras, and in the DWR halo camera the breaks were a simple pass lasting a maximum of 3 min.

Additionally, by studying the profile of human subjects by type of activity (conditions, training, visits/tourism), the rate of improvement of conditions for those previously contaminated with COVID was highlighted, from 7.13% men, the improvement rate was 5.44%; instead, from 8.51% women, the improvement rate was 6.69%.

In the recovery therapies of COVID, cardio–respiratory and immuno–thyroid conditions, the improvement rate had about the same differences between men and women; on the other hand, in the psycho–neuromotor recovery in men, the effect of solion therapy was stronger than in women.

The observations regarding the evolution of the solions in the three halochambers, with static or dynamic regime, respectively, in the conditions with high relative hygroscopicity, correlated with the data related to the stationary period and the treatment regime allowed the development of new artificial (surface) halochambers.

Regarding the strengths of the research, we could mention: the use for the first time of an evaluation in a saltmine in order to determine the optimal values of aerosols, in order to be used after in artificial halochambers and, respectively, a wide range of diseases is presented, which were influenced by the aerosols, with the possibility of treatment.

Regarding the limitations: The study required long time evaluations (our study did continue) and the research involved a large number of patients; also, the saltmine had access to many visitors.

## Figures and Tables

**Table 1:** Applications studied within our group [15].

Medical Domain Application	The Level of Anhydrous Aerosols (mg/m^3^) *	The Level in Solions in the Halochamber (mg/m^3^) **	Composition of Microgranules (g/L) ***	Working Mode of the Device for Generating Solions ****	References
Prevention and treatment of respiratory (airways) disorders	>16	>8.0	NaCl = 280–300	a—73–75 °C;	[1,2,3,19,20,24,25,26,27,29,30,31,32,39,40,41,42,43,44,45,46,47,48,49,50,51,52,53,54,55,56]
				b—0.8–0.9 atm;	
				c—1.1–1.2 atm;	
				d—55–60 °C;	
				e—75–80%UR	
				f—72 h	
Treatment of high blood pressure	>20	>10.0	NaCl = 250–280 KCl = 380–400, MgCl_2_ = 320–350 The ratio: NaCl:KCl:MgCl_2_ = 8:1:1	a—73–75 °C;	[20,29,30,31,32,45,46,47,48,49,50,51,52,53,54,55,56]
				b—0.8–0.9 atm;	
				c—1.2–1.3 atm;	
				d—55–60 °C;	
				e—75–80%UR	
				f—54 h	
Thyroid gland disease therapy	>24	>12.0	NaCl = 250–280 KI = 130–150 The ratio: NaCl:KCl = 9.5:0.5	a—73–75 °C;	[20,29,30,31,32,45,46,47,48,49,50,51,52,53,54,55,56]
				b—0.8–0.9 atm;	
				c—1.2–1.3 atm;	
				d—55–60 °C;	
				e—75–80%UR	
				f—54 h	
Psychomotor disorders	Between 2–12	between 1.0–6.0	NaCl = 230–250 KCl = 380–400 MgCl_2_ = 320–350 CaCl_2_ = 420–450 The ratio: NaCl:KCl:MgCl_2_:CaCl_2_ = 8:1:0.6:0.4	a—73–75 °C;	[20,29,30,31,32,45,46,47,48,49,50,51,52,53,54,55,56,57]
				b—0.8–0.9 atm;	
				c—1.1–1.2 atm;	
				d—50–55 °C;	
				e—55–60%UR	
				f—48 h	
Treating neuro-motor disorders and improving physical performance in children, the elderly and people working in high effort conditions	between 2–12	between 1.0–6.0	NaCl = 230–250 KCl = 380–400 MgCl_2_ = 320–350 CaCl_2_ = 420–450 The ratio: NaCl:KCl:MgCl_2_:CaCl_2_ = 8:1:0.6:0.4	a—73–75 °C;	[20,29,30,31,32,45,46,47,48,49,50,51,52,53,54,55,56,57]
				b—0.8–0.9 atm;	
				c—1.1–1.2 atm;	
				d—50–55 °C;	
				e—55–60%UR	
				f—48 h	
Improving the performance of young athletes	between 1.2–2.0	between 0.6–1.0	NaCl = 250–280 KCl = 380–400 MgCl_2_ = 320–350 KI = 130–150 The ratio: NaCl:KCl:MgCl_2_:KI = 8.5:0.85:0.6:0.05	a—73–75 °C;	[20,29,30,31,32,45,46,47,48,49,50,51,52,53,54,55,56,57,58]
				b—0.8–0.9 atm;	
				c—1.1–1.2 atm;	
				d—50–55 °C;	
				e—55–60%UR	
				f—48 h	
Preventing or stopping the formation of biofilms on the surfaces of prostheses for bone and teeth implants	>16	>8.0	NaCl = 280–300	a—73–75 °C;	[20,29,30,31,32,45,46,47,48,49,50,51,52,53,54,55,56]
				b—0.8–0.9 atm;	
				c—1.1–1.2 atm;	
				d—55–60 °C;	
				e—75–80%UR	
				f—72 h	

* The relative humidity of the environment < 20%. ** The relative humidity of the environment > 80%. *** Microgranules are obtained by hot recrystallization from supersaturated aqueous solutions, followed by loading in dried air diaphragms with humid and warm air vehicles for dispersion in halo-chambers; **** Working mode of the device for generating solions in the halo-chamber: temperature (a) of the supersaturated aqueous solution, depressurization (b) on drying with dried air, the dispersion of the micro-crystallites from the surface efflorescence by blowing (c) hot air (d) and humid air (e), the minimum life time of the solion (f).

**Table 2:** Physico–chemical and microclimate characteristics of the three halochambers: semi-wet static regime (SSR), semi-wet dynamic regime (DSR) and dynamic wet regime (DWR).

Characteristics	Halocahamber SSR			Halochamber DSR			Halochamber DWR		
	Min.	Max.	Average	Min.	Max.	Average	Min.	Max.	Average
Temperature (°C)	12.7	13.5	13.1	12.4	13.2	12.8	12.1	12.5	12.3
Relative humidity (%)	70	74	72	71	77	74	90	96	93
Atmospheric pressure (mmHg)	748	752	750	739	741	740	758	762	760
Light (lx)	88	92	90	79	81	80	104	108	106
Concentration CO_2_ (%)	0.075	0.085	0.08	0.062	0.078	0.07	0.058	0.062	0.06
Concentration O_2_ (%)	20.7	20.9	20.8	20.8	21.0	20.9	20.9	21.2	21.1

**Table 3:** Data regarding the characteristics of solions on the four groups of particles: (1) PM1, (2) PM2.5, (3) PM4, (4) PM10 (1.0, 2.5, 4.0 and 10.0 µm), determined in the three active halochambers of Salina Tg. Ocna (static semi-wet regime—SSR, dynamic semi-wet regime—DSR, and dynamic wet regime—DWR).

The Type of Particles	Halochamber	Measuring Time (s)	Logging Interval Counting Tool (s)	Minimum Aerosol Density (mg/m^3^)	Maximum Aerosol Density (mg/m^3^)	Average Aerosol Density (mg/m^3^)	Observations
PM1	SSR	60	1	0.025	0.068	0.030	The lowest level with very small differences between the concentrations of solions of different sizes
PM2.5	SSR	60	1	0.032	0.047	0.036	
PM4	SSR	60	1	0.036	0.199	0.045	
PM10	SSR	60	1	0.071	0.080	0.047	
PM1	DSR	60	1	0.027	0.067	0.032	A higher level with small differences between the concentrations of solions of different sizes
PM2.5	DSR	60	1	0.040	0.103	0.046	
PM4	DSR	60	1	0.044	0.063	0.049	
PM10	DSR	60	1	0.047	0.102	0.059	
PM1	DWR	60	1	0.041	0.055	0.046	The highest level in solions, with big differences between the four granulometric groups
PM2.5	DWR	60	1	0.057	0.087	0.067	
PM4	DWR	60	1	0.059	0.130	0.099	
PM10	DWR	60	1	0.068	0.185	0.117	

**Table 4:** Profile of human subjects (weight, %), by sex and age groups, correlated with the duration of the work regime in the three halochambers.

Goal	People (%)	Contaminated with COVID, with a Negative Test (%)	Gender and Age Group						Duration (Minutes)		
			Male			Female			SSR	DSR	DWR
			Total (%)	≤35 years (%)	˃35 years (%)	Total (%)	≤35 years (%)	˃35 years (%)			
Treatment	72.99	15.64	31.53	14.35	17.18	41.46	18.56	22.90	1–5	3–6	10–20
Workouts	7.59	1.89	4.22	4.22	-	3.37	3.37	-	1–3 *	10–30 *	2–6 *
Relaxation/visit	19.42	3.03	9.86	4.64	5.22	9.56	4.22	5.34	1–3	3–5	5–10

* Cyclic training regimes, with short breaks of 5–10 min.

**Table 5:** Profile of human subjects by sex and age groups, who performed specific sessions of therapy, training and visit in the three halochambers recommended by the attending physician or other specialized personnel (trainers), the data are represented by the total weight of subjects per group of activities, in correlation with the distribution of subject weight/improvement rate (%) across the three halochambers.

	Type of Halochamber and Group of Subjects	The Working Mode of the Halochamber	Semi-Wet Static Regime (SSR)				Semi-Wet Dynamic Regime (DSR)				Wet Dynamic Regime (DWR)			
Profile of Activities		Activities (%)	Gender				Gender				Gender			
			Male		Female		Male		Female		Male		Female	
			Age Group (Years)		Age Group (Years)		Age Group (Years)		Age Group (Years)		Age Group (Years)		Age Group (Years)	
			˂35	≥35	˂35	≥35	˂35	≥35	˂35	≥35	˂35	≥35	˂35	≥35
Treatments and other activities (Total)		100 ^a^/72.99 ^b^	- ^d^	- ^d^	- ^d^	- ^d^	- ^e^	- ^e^	- ^e^	- ^e^	15.65/9.70 ^f^	19.38/12.02 ^f^	18.56/14.55 ^f^	23.21/18.11 ^f^
COVID recovery		15.64	- ^d^	- ^d^	- ^d^	- ^d^	- ^e^	- ^e^	- ^e^	- ^e^	3.18/2.02 ^f^	3.95/3.42 ^f^	3.78/3.12 ^f^	4.73/3.57 ^f^
Lung disorders		32.82 ^a^/12.8 ^c^	- ^d^	- ^d^	- ^d^	- ^d^	- ^e^	- ^e^	- ^e^	- ^e^	6.69/4.09 ^f^	8.28/5.04 ^f^	7.93/6.34 ^f^	9.92/8.44 ^f^
Heart disorders		13.11 ^a^/4.3 ^c^	- ^d^	- ^d^	- ^d^	- ^d^	- ^e^	- ^e^	- ^e^	- ^e^	2.87/1.87 ^f^	3.31/2.15 ^f^	3.17/2.54 ^f^	3.96/3.57 ^f^
Tyroid and immunity system disorders		6.80 ^a^/1.1 ^c^	- ^d^	- ^d^	- ^d^	- ^d^	- ^e^	- ^e^	- ^e^	- ^e^	1.19/0.82 ^f^	1.72/1.18 ^f^	1.64/0.87 ^f^	2.05/1.68 ^f^
Psycho-neuro-motor recovery		4.62 ^a^/0.2 ^c^	- ^d^	- ^d^	- ^d^	- ^d^	- ^e^	- ^e^	- ^e^	- ^e^	1.72/0.90 ^f^	1.17/0.23 ^f^	1.11/0.75 ^f^	1.40/0.85 ^f^
Sport workouts		7.59 ^a^/1.89 ^c^	4.22	-	3.37	-	- ^e^	- ^e^	- ^e^	- ^e^	- ^g^	- ^g^	- ^g^	- ^g^
Relaxation		19.42 ^a^/3.03 ^c^	4.64	4.22	4.22	2.53	- ^e^	- ^e^	- ^e^	- ^e^	- ^g^	- ^g^	- ^g^	- ^g^

^a^ Total human subjects previously contaminated with COVID, having a negative test obtained in the last 30 days; ^b^ human subjects with conditions; ^c^ human subjects with conditions and who were previously contaminated with COVID, having a negative test obtained in the last 30 days; ^d^ short visits for periods of 3–5 min; ^e^ short visits for periods of 5–10 min; ^f^ the rate of amelioration of the affections (with red color); ^g^ visits for periods of 10–20 min.

**Table 6:** Disease improvement rate (%) following therapy using a complete regimen recommended by the attending physician in the DWR halochamber.

Treatments	Percentage (%)	Men		Women	
		˂35	≥35	˂35	≥35
Therapy cases (Total)	72.99/34.04 ^b^	14.35/9.70 43.20	17.18/12.02	18.56/14.55 56.80	22.90/18.11
COVID recovery	15.64 ^a^	3.18/2.02 45.59	3.95/3.42	3.78/3.12 54.41	4.73/3.57
Lung disorders	31.97/12.8 ^c^	5.32/4.09 38.63	7.03/5.04	8.86/6.34 61.37	10.76/8.44
Heart disorders	13.38/4.3 ^c^	2.94/1.87 46.71	3.31/2.15	3.17/2.54 53.29	3.96/3.57
Diseases of the thyroid and the immune system	6.60/1.1 ^c^	1.19/0.82 44.09	1.72/1.18	1.64/0.87 55.91	2.05/1.68
Psycho-neuro-motor recovery	5.40/0.2 ^c^	1.72/0.90 53.52	1.17/0.23	1.11/0.75 46.48	1.40/0.85

^a^ Subjects previously infected with COVID, having a negative test obtained in the last 30 days; ^b^ human subjects with conditions; ^c^ human subjects with conditions and who were previously contaminated with COVID, having a negative test obtained in the last 30 days.

## Data Availability

All data is presented in article.
